# Comparison of freezing tolerance, compatible solutes and polyamines in geographically diverse collections of *Thellungiella sp.* and *Arabidopsis thaliana* accessions

**DOI:** 10.1186/1471-2229-12-131

**Published:** 2012-08-03

**Authors:** Yang Ping Lee, Alexei Babakov, Bert de Boer, Ellen Zuther, Dirk K Hincha

**Affiliations:** 1 Max-Planck-Institut für Molekulare Pflanzenphysiologie, Am Mühlenberg 1, Potsdam, D-14476, Germany; 2 All-Russia Research Institute of Agricultural Biotechnology RAAS, Timiryazevskaya St. 42, Moscow, 127550, Russia; 3 Department of Structural Biology, Vrije Universiteit Amsterdam, De Boelelaan 1085-1087, Amsterdam, 1081 HV, The Netherlands

**Keywords:** Arabidopsis thaliana, Cold acclimation, Compatible solutes, Freezing tolerance, Natural variation, Polyamines, Thellungiella salsuginea

## Abstract

**Background:**

*Thellungiella* has been proposed as an extremophile alternative to *Arabidopsis* to investigate environmental stress tolerance. However, *Arabidopsis* accessions show large natural variation in their freezing tolerance and here the tolerance ranges of collections of accessions in the two species were compared.

**Results:**

Leaf freezing tolerance of 16 *Thellungiella* accessions was assessed with an electrolyte leakage assay before and after 14 days of cold acclimation at 4°C. Soluble sugars (glucose, fructose, sucrose, raffinose) and free polyamines (putrescine, spermidine, spermine) were quantified by HPLC, proline photometrically. The ranges in nonacclimated freezing tolerance completely overlapped between *Arabidopsis* and *Thellungiella*. After cold acclimation, some *Thellungiella* accessions were more freezing tolerant than any *Arabidopsis* accessions. Acclimated freezing tolerance was correlated with sucrose levels in both species, but raffinose accumulation was lower in *Thellungiella* and only correlated with freezing tolerance in *Arabidopsis*. The reverse was true for leaf proline contents. Polyamine levels were generally similar between the species. Only spermine content was higher in nonacclimated *Thellungiella* plants, but decreased during acclimation and was negatively correlated with freezing tolerance.

**Conclusion:**

*Thellungiella* is not an extremophile with regard to freezing tolerance, but some accessions significantly expand the range present in *Arabidopsis*. The metabolite data indicate different metabolic adaptation strategies between the species.

## Background

Low temperatures and freezing impose major limitations on plant growth and development and limit the productivity of crop plants in large parts of the world. Plants from temperate regions increase in freezing tolerance during exposure to low but nonfreezing temperatures for a period of days to weeks, a process termed cold acclimation. This is accompanied by massive changes in gene expression and metabolite composition [1-3], including increased levels of compatible solutes such as sugars, proline and polyamines that potentially contribute to cellular freezing tolerance.

The majority of molecular studies of plant freezing tolerance and cold acclimation have been performed in *Arabidopsis thaliana*. In addition to forward and reverse genetics, the analysis of natural variation has become an increasingly useful approach in the analysis of complex adaptive traits in this species (see [4-6] for reviews). *Arabidopsis* accessions are widely distributed throughout the Northern hemisphere, spanning diverse growth environments. It can therefore be expected that they harbour phenotypic and genetic variation that is advantageous for adaptation to various climatic conditions. Several studies have shown significant natural variation in the responses of *Arabidopsis* accessions to low temperature [7-13]. However, *Arabidopsis* is not an extremophile and it could be expected that more freezing tolerant species have evolved different or additional protective mechanisms that cannot be found in this species.

*Thellungiella salsuginea* is an emerging plant model species that has been suggested to possess the characteristics of an extremophile, i.e. high tolerance of salinity, freezing, nitrogen-deficiency and drought stress [14-19]. The genus *Thellungiella* is part of the *Brassicaceae* family and therefore related to *Arabidopsis thaliana*[20,21]. *T. salsuginea* resembles *Arabidopsis* in many features such as short life cycle, self-fertility, transformation by the floral-dip method and a genome size approximately twice that of *Arabidopsis*[17]. The genome of the closely related species *T. parvula* has recently been sequenced [22]. Similar to *Arabidopsis*, also in *T. salsuginea* different accessions have been identified and the Shandong and Yukon accessions, which originate from China and Canada, respectively, have frequently been used to investigate responses to abiotic stresses [21]. However, no systematic investigation of natural variation in the stress tolerance of *Thellungiella* has been published to date.

Here we present such a study, investigating the freezing tolerance and cold acclimation responses of 14 *T. salsuginea* accessions and of the two closely related species *T. halophila* and *T. botschantzevii*. We compare these data to the results of a recent study on 54 *Arabidopsis* accessions [13]. Our results suggest that the freezing tolerance after cold acclimation of the *Thellungiella* accessions extends to lower temperatures than the freezing tolerance of the most tolerant *Arabidopsis* accessions. In addition, the data provide the first evidence for a different metabolic acclimation strategy in *Thellungiella* compared to *Arabidopsis*.

## Methods

### Plant material

Seeds of the *Thellungiella salsuginea* ((Pallas) O.E. Schulz) accessions Colorado, Cracker Creek, Dillibrough, Hebei, Henan, Jiangsu, Shandong, Xinjiang and Yukon were kindly provided by Prof. Ray A. Bressan (Purdue University, West Lafayette, IN). Seeds of further *T. salsuginea* accessions (Altai 1, Altai 2, Buriatia, Tuva and Yakutsk), *T. halophila* ((C.A. Meyer) O.E. Schulz) (Bayanaul) and *T. botschantzevii* (D.A. German) (Saratov) were collected in Russia and Kazakhstan. The geographical origins of all accessions are listed in Table 1. The *A. thaliana* accessions used for polyamine determination are those used in our previous studies [7,13].

**Table 1:** ** *Thellungiella* accessions with information on their geographic origins**

**Accession**	**Species**	**Origin**^**a**^	**Latitude**	**Longitude**	**Min. Temp. (°C)**^**c**^
**Saratov**	*Thellungiella botschantzevii*	Flood-lands of Kurdium river, Saratov Region, Russian Federation	51°N	45-46°E	−6
**Bayanaul**	*Thellungiella halophila*	Pavlodar Region, Kazakhstan	50°47'N	75°42'E	−15
**Altai 1**	*Thellungiella salsuginea*	2000 m height near Kosh Agach plateau, Russian Federation	49°59'19"N	88°40'19"E	−18
**Altai 2**	*Thellungiella salsuginea*	2000 m height near Kosh Agach plateau, Russian Federation. About 1 km apart from Altai 1	49°59'19"N	88°40'19"E	−18
**Buriatia**	*Thellungiella salsuginea*	Buryatia Republic, Russian Federation	51-55°N	NA^b^	
**Colorado**	*Thellungiella salsuginea*	Park County, Colorado, USA	39°7'12"N	105°42'36"W	−7
**Cracker Creek**	*Thellungiella salsuginea*	Cracker Creek, British Columbia, Canada	59°42'N	133°24'W	−11
**Dillibrough**	*Thellungiella salsuginea*	Unknown	NA^b^	NA^b^	
**Hebei**	*Thellungiella salsuginea*	High saline-alkaline wasteland at Fengnan District, Hebei Province, China	39°20'24"N^d^	118°3'36"E^d^	0
**Henan**	*Thellungiella salsuginea*	Near wheat field at Xinxiang, Henan Province, China	35°10'48"N^d^	113°31'12"E^d^	3
**Jiangsu**	*Thellungiella salsuginea*	Near saltworks at Sheyang County, Jiangsu Province, China	33°34'48"N^d^	120°33'E^d^	1
**Shandong**	*Thellungiella salsuginea*	Near mouth of Yellow River, Dongying, Shandong Province, China	37°16'12"N^d^	118°18'E^d^	1
**Tuva**	*Thellungiella salsuginea*	Tuva Republic, Russian Federation	51-55°N	NA^b^	−16
**Xinjiang**	*Thellungiella salsuginea*	Near wheat field at Manasi County, Xinjiang Province, China	44°10'48"N^d^	86°18'36"E ^d^	−5
**Yakutsk**	*Thellungiella salsuginea*	Yakutsk, Sakha Republic, Russian Federation	61°N	130°E	−26
**Yukon**	*Thellungiella salsuginea*	Takhini Salt Flats, Yukon Territory, Canada	60°51'17"N	135°43'2"W	−11

^a^Origin of the collection site.

^b^NA, not available.

^c^Average minimum habitat temperature recorded during the coldest month of the growing season (March to October) at the recording station nearest to the collection site (http://www.weatherbase.com and http://weather.za.msn.com).

^d^Latitude and longitude of the collection sites is extracted as described [23].

Seeds of the *Thellungiella* accessions were sown in soil and exposed to 4°C in a growth cabinet at 16 h day length with 90 μE m^-2^ s^-1^ for one week to promote germination. Seedlings were transferred to a greenhouse at 16 h day length with light supplementation to reach at least 200 μE m^-2^ s^-1^ at a temperature of 20°C during the day and 18°C during the night for 8 weeks (nonacclimated plants). For cold acclimation, plants were transferred to a 4°C growth cabinet under the conditions described above for an additional 14 days. *Arabidopsis* plants were grown and acclimated under identical conditions [7,11], but were only grown under nonacclimating conditions for 6 weeks to reach the same developmental state.

### Freezing tolerance assays

Freezing damage was determined as electrolyte leakage after freezing of detached leaves to different temperatures as described in detail in previous publications [7,11]. Briefly, series consisting of three rosette leaves taken from three individual plants were placed in glass tubes containing 300 μl of distilled water. The tubes were transferred to a programmable cooling bath set to −1°C, control samples were left on ice during the entire experiment. After 30 min of temperature equilibration at −1°C, ice crystals were added to the tubes to initiate freezing. After another 30 min, the samples were cooled at a rate of 4°C/h. Over a temperature range of −1°C to −30°C samples were taken from the bath and thawed slowly on ice over night. Leaves were then immersed in distilled water and placed on a shaker for 16 h at 4°C. Electrolyte leakage was determined as the ratio of conductivity measured in the water before and after boiling the samples. The temperature of 50% electrolyte leakage (LT_50_) was calculated as the LOG EC_50_ value of sigmoidal curves fitted to the leakage values using the software GraphPad Prism3.

### Sugar analysis

Two leaves from plants that were also used in the freezing tolerance assays were frozen in liquid nitrogen immediately after sampling and homogenized using a ball mill “Retsch MM 200” (Retsch, Haan, Germany). Soluble sugars were extracted and quantified by high performance anion exchange chromatography (HPAEC) using a CarboPac PA-100 column on an ICS3000 chromatography system (Dionex, Sunnyvale, CA) as described previously [24].

### Proline measurements

Proline content was measured from the ethanolic extracts that were also used for sugar determination following a method modified from a previously described procedure [25,26]. The extracts were diluted 10-fold with distilled water and 100 μl were combined with 100 μl of glacial acetic acid and 100 μl of 2.5% (w/v) acid ninhydrine reagent [26]. The mixture was incubated at 95°C for 1 h and then for 10 min on ice. The reaction mixture was extracted with 500 μl of toluene and the ninhydrine absorbance was measured in the toluene phase at 520 nm in a spectrophotometer.

### Polyamine measurements

Leaf samples (100–200 mg) were homogenized with a ball mill, extracted in 1 ml of 0.2 N perchloric acid for 1 h at 4°C to extract free polyamines and centrifuged at 16000 x g at 4°C for 30 min. Since we detected only very low levels of bound polyamines in our samples (data not shown), these were not further investigated. To 100 μl aliquots of the supernatants, 110 μl of 1.5 M sodium carbonate and 200 μl of dansyl chloride (7.5 mg/ml in acetone; Sigma, Munich, Germany) were added. In addition, 10 μl of 0.5 mM diaminohexane were added as an internal standard. After 1 h incubation at 60°C in the dark, 50 μl of a 100 mg/ml proline solution was added to bind free dansyl chloride [27]. After 30 min incubation at 60°C in the dark, dansylated polyamines were extracted with 250 μl toluene, dried in a vacuum centrifuge and dissolved in 100 μl methanol. Analyses were performed with a reverse phase LC-18 column (Supelco, Munich, Germany) on a HPLC system (Dionex) consisting of a gradient pump (model P 580), an automated sample injector (ASI-100) and a fluorescence detector (RF 2000). Twenty μl samples were injected, polyamines were eluted with a linear gradient of from 70% to 100% (v/v) methanol in water at a flow rate of 1 ml/min and detected at an excitation wavelength of 365 nm and an emission wavelength of 510 nm. Data were analyzed using the Dionex Chromeleon software and quantification was performed with calibration curves obtained from the pure substances.

### Statistics

Correlation tests were performed using Pearson's product–moment correlation analysis in the R statistics package [28].

## Results

### *Establishment of a collection of natural* Thellungiella spec. *accessions*

We investigated the cold acclimation and freezing tolerance of 16 different *Thellungiella* accessions (Table 1). Of these, 14 belong to the species *T. salsuginea* and one each to *T. halophila* (Bayanaul) and *T. botschantzevii* (Saratov). Four of the accessions originate from the continental USA or Canada and five from China and substantial work has been performed previously on the accessions Yukon and Shandong (see [21] for a review). In addition, seven accessions were collected for this study from different sites in Russia and Kazakhastan to enrich our collection for accessions from very cold climates (Table 1). Thus the geographical origins of these accessions span the Northern hemisphere (between 33°N and 61°N) from 130°E to 135°W.

### *Natural variation in the freezing tolerance of* Thellungiella *accessions*

**Figure 1:**
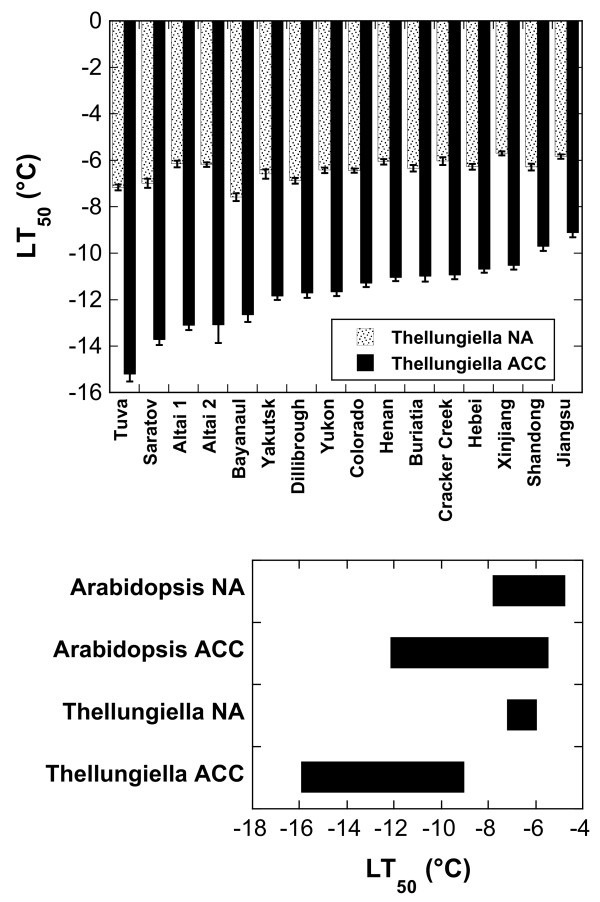
**Freezing tolerance of leaves from 16 *Thellungiella* accessions before (NA) and after (ACC) 14 days of cold acclimation at 4°C.** Freezing tolerance was measured with an electrolyte leakage assay and is expressed as the LT_50_, i.e. the temperature that resulted in 50% ion leakage from the leaves. All accessions and information on their geographical origins are listed in Table 1. The bars in the top panel represent the means ± SE from five replicate measurements where each replicate comprised leaves from three plants. The accessions are ordered from the lowest LT_50_ after cold acclimation on the left to the highest on the right. The bottom panel shows the range of LT_50_ values before and after cold acclimation for 54 *Arabidopsis* accessions [13] and the 16 *Thellungiella* accessions investigated in the present study.

The freezing tolerance of the *Thellungiella* accessions was determined before (nonacclimated; NA) and after (acclimated; ACC) two weeks of cold acclimation at 4°C (Figure 1). The results show strong natural variation in the freezing tolerance of *Thellungiella*. Higher variation was found in acclimated (LT_50_ from −9.12°C (Jiangsu) to −15.21°C (Tuva)), than in nonacclimated plants (LT_50_ from −5.70°C (Xinjiang) to −7.40°C (Bayanaul)). In addition, Tuva showed the highest acclimation capacity (8.22°C difference in LT_50_ between NA and ACC plants) and Jiangsu the lowest (3.28°C).

*Thellungiella* is generally considered to be much more freezing tolerant than Arabidopsis [29]. The fact that we have recently determined the freezing tolerance of 54 *Arabidopsis* accessions under exactly the same conditions as used here for *Thellungiella*[13] provided a unique opportunity to test this assumption. Figure 1 clearly shows that the range of LT_50_ values was not different between *Arabidopsis* and *Thellungiella* in the nonacclimated state, but that some *Thellungiella* accessions (Tuva, Saratov, Altai 1 and 2, Bayanaul) reached lower LT_50_ values after cold acclimation.

**Figure 2:**
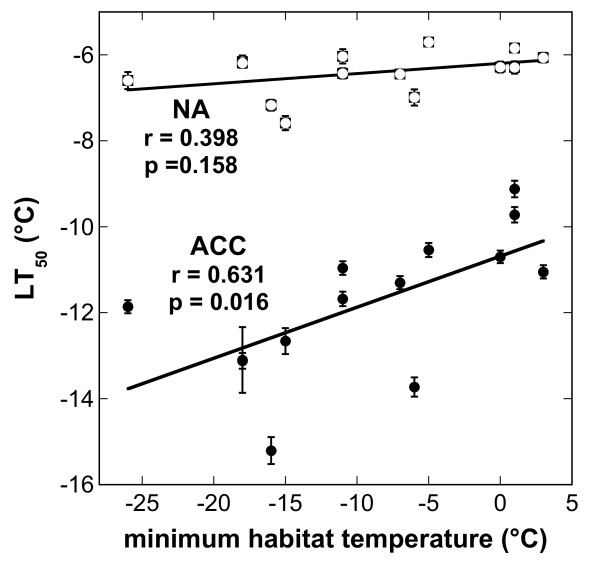
**Correlation between the average minimum habitat temperature recorded during the coldest month of the growth season (Table**1**) and the LT**_**50**_**of the leaves from either nonacclimated (NA) or cold acclimated plants (ACC).** The lines were fitted to the data by linear regression analysis and the correlation coefficients and p-values are shown in the figure.

No significant correlations at p < 0.05 were found between the latitude of the geographical origin of the accessions and their LT_50_ either before or after cold acclimation. However, LT_50_ ACC was significantly correlated with the average minimum habitat temperature recorded during the coldest month of the growth season, while no such correlation was found before cold acclimation (Figure 2).

### Accumulation of sugars and proline in response to cold

**Figure 3:**
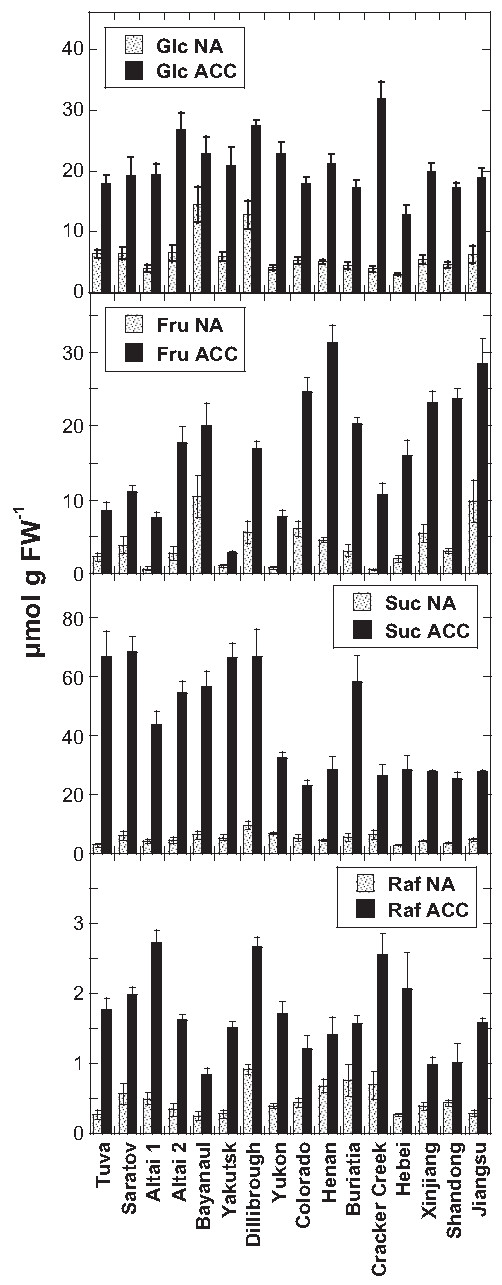
**Contents of soluble sugars in the leaves of all investigated *Thellungiella* accessions.** Leaves were harvested either before (NA) or after (ACC) cold acclimation. Note the different scales of the ordinates in the different panels. The accessions are ordered from the lowest LT_50_ after cold acclimation on the left to the highest on the right. The bars represent means ± SE from measurements of seven to 10 samples from two independent experiments.

**Figure 4:**
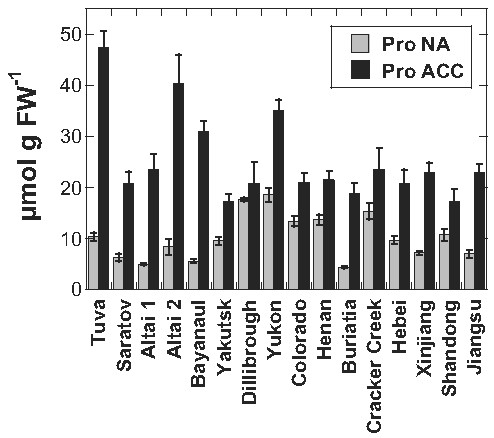
**Proline contents in the leaves of all investigated *Thellungiella* accessions.** Leaves were harvested either before (NA) or after (ACC) cold acclimation. The accessions are ordered from the lowest LT_50_ after cold acclimation on the left to the highest on the right. The bars represent means ± SE from measurements of nine or 10 samples from two independent experiments.

The accumulation of compatible solutes such as sugars and proline is commonly observed during cold acclimation [2,3]. We therefore measured the amounts of glucose (Glc), fructose (Fru), sucrose (Suc), raffinose (Raf) and proline (Pro). Figures 3 and 4 show that the contents of sugars and Pro increased strongly in leaf samples of most *Thellungiella* accessions during cold acclimation. As observed previously for *Arabidopsis*[13], there were also some *Thellungiella* accessions that failed to accumulate a particular solute. For instance, Yakutsk showed an extremely low level of Fru after acclimation, while Dillibrough did not accumulate any Pro in the cold.

**Figure 5:**
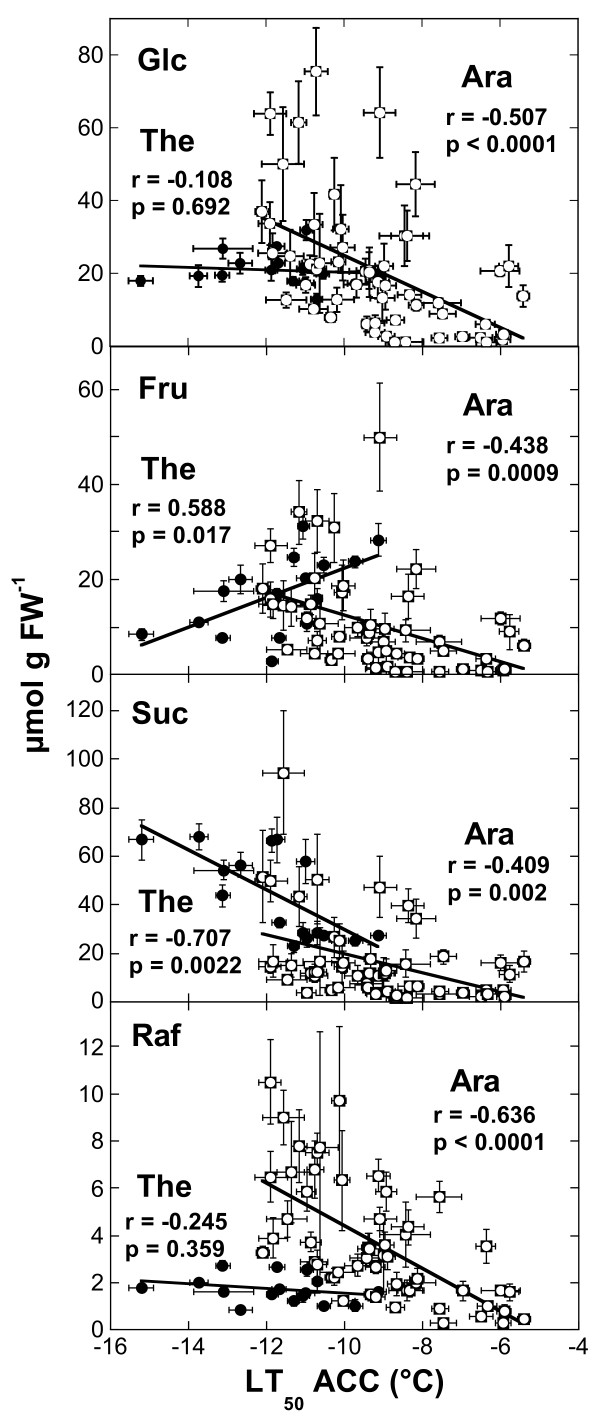
**Correlations among the contents of different soluble sugars in *Arabidopsis* and *Thellungiella* and their freezing tolerance after cold acclimation.** The lines were fitted to the data by linear regression analysis and the correlation coefficients and p-values are shown in the panels. The data for *Thellungiella* (solid symbols) are the same as those shown in Fig. 1 for LT_50_ and in Fig. 3 for sugar contents. The data for *Arabidopsis* (open symbols) are taken from [13].

**Figure 6:**
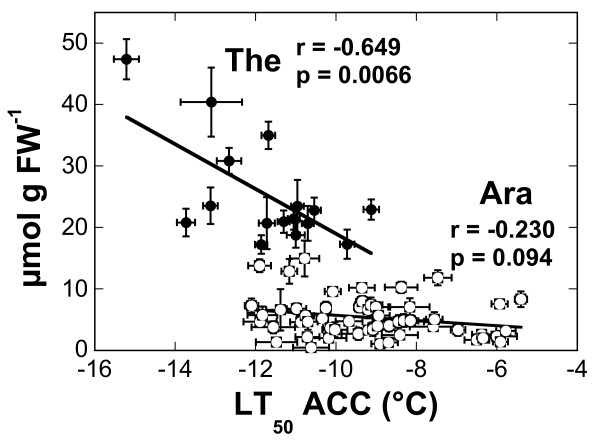
**Correlations between the proline content of *Arabidopsis* and *Thellungiella* leaves and their freezing tolerance after cold acclimation.** The lines were fitted to the data by linear regression analysis and the correlation coefficients and p-values are shown in the panels. The data for *Thellungiella* (solid symbols) are the same as those shown in Fig. 1 for LT_50_ and in Fig. 4 for proline content. The data for *Arabidopsis* (open symbols) are taken from [13].

We further explored the functional significance of these compatible solutes in leaf freezing tolerance by correlation analysis. The contents of sugars and Pro were not significantly correlated with LT_50_ under nonacclimated condition except for Glc (r = −0.619, p = 0.011). After cold acclimation, only the contents of Suc (Figure 5) and Pro (Figure 6) were significantly positively correlated with freezing tolerance (i.e. negative correlation with LT_50_), while the content of Fru was negatively correlated. In other words, the contents of Suc and Pro were higher in the more freezing tolerant accessions, while the contents of Fru was higher in the more sensitive accessions.

Since we had previously also determined the sugar and Pro contents of the leaves of 54 *Arabidopsis* accessions [13], we could now directly compare the role of compatible solutes in the acclimated freezing tolerance of these species (Figures 5 and 6). While Glc, Fru and Suc contents were significantly positively correlated with freezing tolerance in *Arabidopsis*, this was only true for Suc in *Thellungiella*. However, the overall pool sizes of these sugars were similar, although some *Arabidopsis* accessions accumulated two- to three-fold higher amounts of Glc. The most striking differences were found for Raf and Pro. The amounts of Raf in the leaves of the most freezing tolerant acclimated *Arabidopsis* accessions were several-fold higher than those of any *Thellungiella* accessions. For example, the most freezing tolerant *Arabidopsis* accession (N14) contained about 10.5 μmol Raf g^-1^ FW, while all *Thellungiella* accessions accumulated less than 3 μmol g^-1^ FW. On the other hand, Pro levels were much higher in *Thellungiella* than in *Arabidopsis* leaves and there was no significant correlation between Pro contents and LT_50_ ACC in *Arabidopsis* (Figure 6). Some *Thellungiella* accessions already contained more Pro in their leaves in the nonacclimated state (up to 18.5 μmol g FW^-1^) than any *Arabidopsis* accession after cold acclimation (up to 14.9 μmol g FW^-1^).

### *Polyamine contents in* Thellungiella *and* Arabidopsis *accessions*

**Figure 7:**
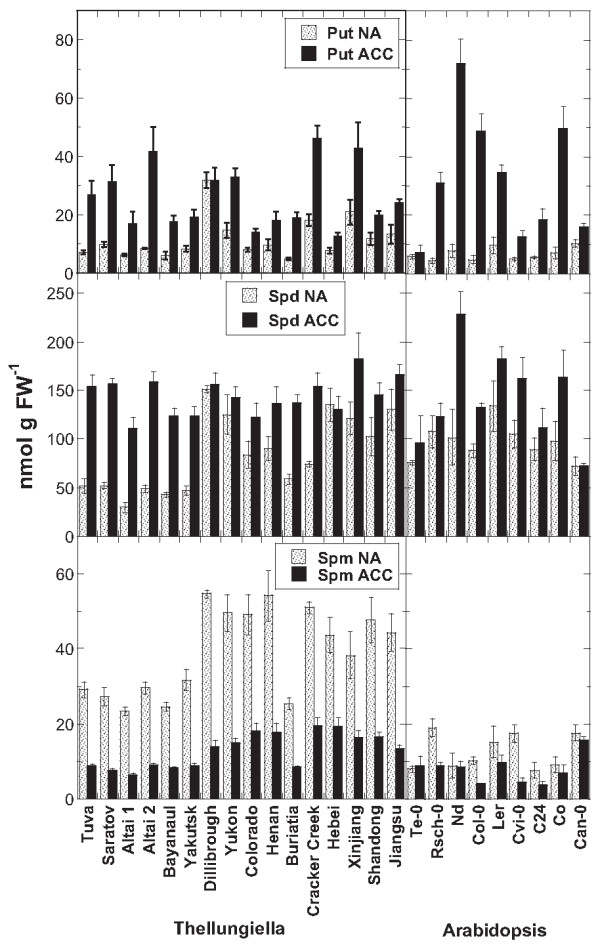
**Contents of soluble polyamines in the leaves of all investigated *Thellungiella* and nine *Arabidopsis* accessions.** Leaves were harvested either before (NA) or after (ACC) cold acclimation. Note the different scales of the ordinates in the different panels. The accessions are ordered from the lowest LT_50_ after cold acclimation on the left to the highest on the right separately for *Thellungiella* and *Arabidopsis*. The bars represent means ± SE from measurements of eight to 10 samples from two independent experiments for *Thellungiella* and three samples from one experiment for *Arabidopsis*.

There is evidence from several studies that polyamines may play important roles in the development of plant freezing tolerance (see [30] for a recent review). We have therefore measured the amounts of free putrescine (Put), spermidine (Spd) and spermine (Spm) in leaf samples from all *Thellungiella* accessions both before and after cold acclimation (Figure 7). Since no published data on the polyamine contents of different *Arabidopsis* accessions under these conditions were available, we also determined the respective polyamines in nine Arabidopsis accessions that span a wide range of freezing tolerance [7,13]. In general, the levels of Put and Spd were similar in *Thellungiella* and *Arabidopsis* and they either increased during cold acclimation or remained unaltered in some accessions (e.g. Dillibrough and Hebei; Te-0 and Can-0). However, the levels of Spm were much higher in nonacclimated *Thellungiella* leaves, but were drastically reduced during cold acclimation. In *Arabidopsis*, Spm levels were generally lower and only decreased in a few accessions during acclimation. In both species free Spd was the predominant polyamine under both conditions.

**Figure 8:**
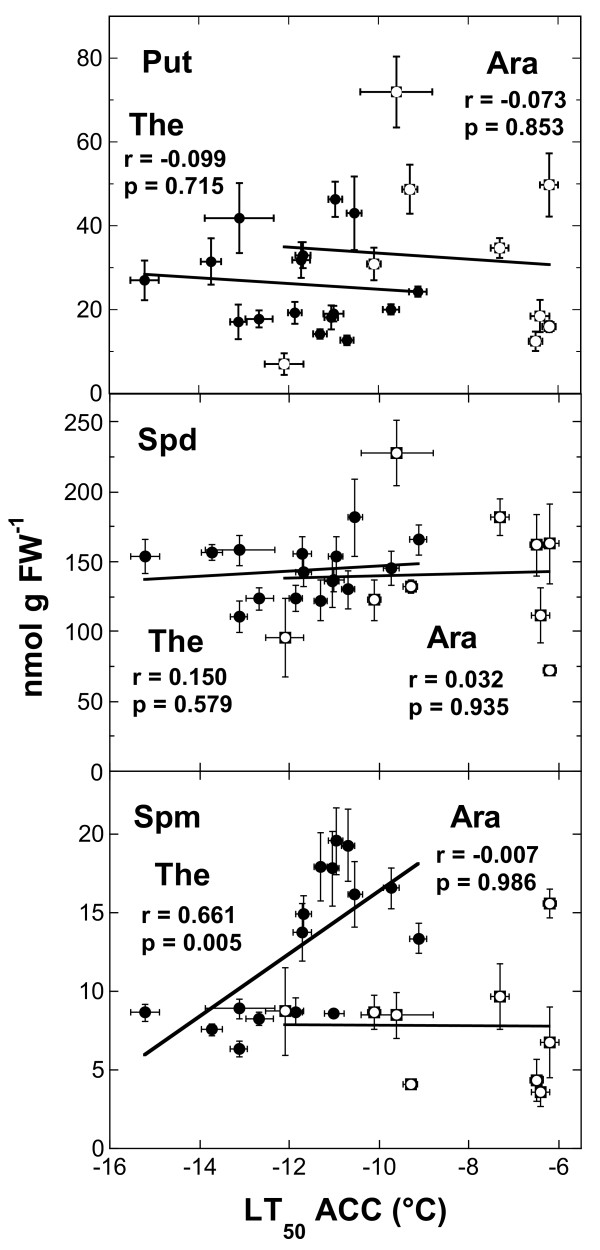
**Correlations among the contents of different soluble polyamines in the *Thellungiella* and *Arabidopsis* accessions and their freezing tolerance after cold acclimation.** The lines were fitted to the data by linear regression analysis and the correlation coefficients and p-values are shown in the panels. Solid symbols denote data from *Thellungiella*, open symbols data from *Arabidopsis*.

No significant correlations were found among the Put, Spd or Spm contents and LT_50_ NA (not shown) or Put or Spd contents and LT_50_ ACC in *Thellungiella* (Figure 8). However, there was a significant correlation between Spm content and LT_50_ ACC in *Thellungiella*, indicating that higher leaf freezing tolerance was correlated with a lower pool size of free Spm. In *Arabidopsis*, no correlations among LT_50_ and polyamine pool sizes were observed under either condition.

## Discussion

*Thellungiella* has been proposed as an alternative model species to *Arabidopsi*s to investigate plant abiotic stress tolerance mechanisms. *Thellungiella* shares many features with *Arabidopsis* that make it an attractive candidate for both physiological and molecular studies [14,21,29]. The main argument in favor of *Thellungiella*, however, is that it is considered an “extremophile” that is much more tolerant to various stresses than *Arabidopsis*. On the other hand, it has been shown that there is considerable natural variation between different accessions of *Arabidopsis* that results in different levels of tolerance under various environmental growth and stress conditions (see e.g. [6] for a recent review). This natural variation has been investigated most extensively for cold acclimation and freezing tolerance [7,8,10,12,13]. Since natural accessions are also available for *Thellungiella* this opens the unique possibility to directly compare the range of stress tolerance and possible differences in adaptive mechanisms between these species.

In the present study, we have for the first time compared the range of natural variation in the freezing tolerance of *Arabidopsis* and *Thellungiella*. We conclude from the wide overlap in the freezing tolerance that at least with regard to this trait *Thellungiella* should not be considered an extremophile. Its range of freezing tolerance, however, extends to lower temperatures than that of *Arabidopsis* with about one-third of the available *Thellungiella* accessions more freezing tolerant than any *Arabidopsis* accession. The acclimated freezing tolerance of *Thellungiella* was positively correlated with the average minimum habitat temperature recorded during the coldest month of the growth season, consistent with previous results for *Arabidopsis*[7,12].

Only the freezing tolerance of the Yukon accession of *Thellungiella* has previously been reported in the literature [16]. LT_50_ values of −13°C for nonacclimated and −18.5°C for cold acclimated plants were recorded when whole-plant survival was evaluated. These temperatures are substantially lower than the −6.4°C (NA) and −11.7°C (ACC) obtained from our electrolyte leakage measurements. However, corresponding electrolyte leakage data in [16] suggest a similar temperature range to our results although no LT_50_ values were given. In addition, since no direct comparison with *Arabidopsis* was presented, any comparison between the species remained speculative in this paper.

From the comparison presented here we suggest that although *Thellungiella* may not be an extremophile with regard to freezing tolerance, its range of freezing tolerance after cold acclimation clearly extends beyond *Arabidopsis*. We therefore consider *Thellungiella* a useful additional model species to identify superior or alternative freezing tolerance mechanisms.

During cold acclimation in *Arabidopsis*, the composition of the metabolome is strongly changed (see [1] for a review). The pool sizes of several metabolites are increased and there are significant differences in the cold-responsive metabolomes of different *Arabidopsis* accessions [7,31,32]. Significantly, the leaf contents of the four sugars Glc, Fru, Suc and Raf were linearly correlated with leaf freezing tolerance [8,11,13] and these sugars were also found among a small group of metabolites that could be used to predict the freezing tolerance of several *Arabidopsis* genotypes with high accuracy [32]. In addition, although the Pro contents of the leaves also increased during cold acclimation, there was no correlation with freezing tolerance among the 54 accessions investigated previously [13] and Pro was also not among the predictive metabolites [32].

The present data suggest that the role of these five compatible solutes may be significantly different between *Arabidopsis* and *Thellungiella*. Among the sugars, a positive correlation with acclimated freezing tolerance was only observed for Suc, while there was actually a negative correlation for Fru. In addition, the *Thellungiella* accessions did not accumulate Raf to the same extent as *Arabidopsi*s. Instead, *Thellungiella* accumulated much higher amounts of Pro during cold acclimation and we found a significant correlation with acclimated freezing tolerance. The accumulation of compatible solutes, particularly Suc and Pro, was not only found in *Thellungiella* plants during cold acclimation. Especially Pro contents also increased much more than in *Arabidopsis* when plants were challenged with high NaCl concentrations [15,33,34] suggesting a different metabolic adaptation strategy between the species under abiotic stress conditions. Obviously, this hypothesis has to be tested in the future by metabolomic approaches using appropriate collections of accessions from both species.

We would like to stress at this point that it is highly unlikely that the differences in compatible solute content are the only reason for the observed differences in freezing tolerance. Although the constitutively freezing tolerant *esk1* mutant in *Arabidopsis* shows a high accumulation of Pro under nonacclimated conditions [35], it also shows hundreds of changes in gene expression, making it impossible to attribute the higher freezing tolerance to a single factor [36]. Similarly, although freezing tolerance in *Arabidopsis* is strongly correlated with Raf content, a knock-out mutant of the raffinose synthase gene in Col-0 resulted in the absence of Raf in the cold acclimated leaves without an impairment of freezing tolerance [37]. All these findings emphasize the well-known fact that plant freezing tolerance is a multigenic, quantitative trait. In addition, the present data indicate that even in closely related species, different metabolites may be important.

One additional class of metabolites that has frequently been implicated in plant freezing tolerance are polyamines [30]. They are thought to be involved in many aspects of plant growth, development and stress tolerance (see [38-40] for reviews). Their exact functions in these processes have not been completely elucidated, but it was demonstrated that Put is an essential component of the cold acclimation process in *Arabidopsis*[41]. This is at least in part mediated through a role in the regulation of ABA biosynthesis.

The measurement of free polyamine levels in several accessions of both *Arabidopsis* and *Thellungiella* revealed that not all accessions showed an increase in the content of Put or Spd during cold acclimation. Also, the levels of free Put and Spd were not correlated with leaf freezing tolerance. In fact, the most freezing tolerant *Arabidopsis* accession in this study (Te-0) showed no increase in the pool size of either polyamine. In addition, the overall amounts of Put and Spd were very similar in all studied plants. Only the contents of free Spm showed higher levels in *Thellungiella* under nonacclimating conditions than in *Arabidopsis*. This was, however, strongly decreased during cold acclimation, leading to similar pool sizes between the species in the acclimated state. In *Thellungiella* we found a negative correlation between Spm contents and LT_50_ ACC, indicating that low levels of Spm may be a requirement for efficient cold acclimation. A similar reduction of Spm levels was previously already observed in the *Arabidopsis* accession Col-0 [41] and in wheat [42] in response to cold exposure. However, the functional relevance of this reduction of free Spm levels is currently unknown. The natural variation in Spm content revealed in this study may offer an interesting possibility to elucidate the molecular basis and functional significance of this phenomenon.

## Conclusion

While *Thellungiella* is generally assumed to be an extremophile with regard to its abiotic stress tolerance, the presented data indicate that this is not true with regard to its freezing tolerance. Some accessions, however, significantly expand the range present in *Arabidopsis*, stressing the utility of *Thellungiella* as an additional model species. The metabolite data indicate different metabolic adaptation strategies between these rather closely related species that need to be followed up with appropriate profiling technologies.

## Competing interests

The authors declare that they have no competing interests.

## Authors´ contributions

YPL carried out the freezing tolerance experiments and the proline measurements, YPL and EZ performed the sugar and polyamine determinations. AB and BdB collected and provided *Thellungiella* seeds. YPL, EZ and DKH designed the study and analyzed the data. YPL and DKH drafted the manuscript. All authors read and approved the manuscript.
